# Distribution of *Phasmarhabditis* (Nematode: Rhabditidae) and Their Gastropod Hosts in California Plant Nurseries and Garden Centers

**DOI:** 10.3389/fpls.2022.856863

**Published:** 2022-05-17

**Authors:** Jacob Schurkman, Irma Tandingan De Ley, Kyle Anesko, Timothy Paine, Rory Mc Donnell, Adler R. Dillman

**Affiliations:** ^1^Department of Nematology, University of California, Riverside, Riverside, CA, United States; ^2^Department of Entomology, University of California, Riverside, Riverside, CA, United States; ^3^Department of Crop and Soil Science, Oregon State University, Corvallis, OR, United States

**Keywords:** *Phasmarhabditis californica*, *P. hermaphrodita*, *P. papillosa*, invasive gastropods, nurseries

## Abstract

Three species of *Phasmarhabditis* were recovered from 75 nurseries and garden centers in 28 counties in California during fall and winter 2012–2021. A total of 18 mollusk species were recovered, most of them invasive. Nematodes were identified by sequencing the D2-D3 expansion segments of the large subunit (LSU or 28S) rRNA. Based on these surveys, *P. californica* was the most widespread species (37 isolates, 53.6% recovery); followed by *P. hermaphrodita* (26 isolates; 37.7% recovery); *P. papillosa* and a closely related *P. papillosa* isolate (6 isolates; 8.7% recovery). Nematode isolates were mainly collected from four invasive slugs (*Deroceras reticulatum*, *D. laeve*, *Arion hortensis* agg, *Ambigolimax valentianus*) and snails (*Oxychilus* spp. and *Discus* spp.). Results suggest that *P. californica* and *P. hermaphrodita* share an ecological niche in Northern, Central, Coastal, and Southern California, north of Los Angeles County.

## Introduction

The United States harbors a significant diversity of invasive species (Pimentel et al., 2005). They serve as a threat to the country’s natural biodiversity since the introduction of invasive species is one of the leading causes of global biodiversity decline (Mckinney and Lockwood, 1999; Clavero and Garcia-Berthou, 2005; Butchart et al., 2010; Gladstone and Bordeau, 2020). While the distribution of numerous invasives have been tracked, some taxonomic groups have been largely neglected, notably terrestrial gastropod species, many species of which are of agricultural and horticultural interest (Barker, 2002; Pyšek et al., 2008; Lowry et al., 2012; Gladstone and Bordeau, 2020).

Many invasive terrestrial gastropods in the United States are present on the west coast, especially in California, Oregon, Washington, and Hawaii, where most gastropod surveys have been conducted. For example, in California, it is estimated that there are approximately 279 species of terrestrial gastropods, 37 of which are invasive (Roth and Sadeghian, 2003). These gastropods were likely introduced via the horticultural trade when gastropods residing on plant products were delivered to western states (Cowie et al., 2008; Bergey et al., 2014).

Some of these introduced gastropods are considered among the most pestiferous slugs and snails. These species include *Deroceras reticulatum* (Müller 1774), *Arion hortensis* (Ferrusac 1819), and *Cornu aspersum* (Müller 1774) (Mc Donnell et al., 2009). For example, *C. aspersum* can reduce some California citrus fruit crop yields by 40–50% and occasionally up to 90–100% in years of high rainfall (Pappas and Carman, 1961; Sakovich, 2002). Terrestrial gastropods do not only cause direct physical damage to plants, but they can also spread disease. They have been found to serve as vectors for pathogens like *Alternaria brassicicola* (Saccardo 1880), and members of the family Peronosporaceae, and other plant-pathogenic fungi (Wester et al., 1964; Hasan and Vago, 1966; Turchetti and Chelazzi, 1984). Some gastropod species have also been found to harbor human pathogens. It has been postulated that some slugs and snails have been partially responsible for spinach and other salad crop recalls due to the discovery of *Campylobacter* spp. and *Escherichia coli* (Migula 1895) in the feces of sampled gastropods (Sproston et al., 2006; Raloff, 2007). Multiple terrestrial gastropods have also been found to carry *Angiostrongylus cantonensis* (Chen 1935), the causative agent for eosinophilic meningitis (Lindo et al., 2004; Teem et al., 2013; Iwanowicz et al., 2015).

Invasive terrestrial gastropods can also be detrimental to sensitive ecosystems. Multiple wetlands and marshes are threatened by invasive gastropod species because they thrive in these environments with a lack of natural predators (Cowie, 1998; Silliman et al., 2005). The invasive gastropods are capable of quick propagation and can reach large populations within a relatively short period of time. These large invasive populations result in a lack of resources for other endemic organisms which cannot compete with the invasives (Cowie, 1998; Silliman et al., 2005). Additionally, some native snail species have disappeared due to the introduction of the carnivorous snail *Euglandia rosea* (Ferrusac, 1821) (Cowie, 1998; Silliman et al., 2005). Horticultural and agricultural trade across the world brings danger to endemic organisms. To prevent invasive gastropods from being introduced through horticultural and agricultural trade, effective methods of pest control must be utilized.

The most common method of gastropod pest control is the use of molluscicides. One of the most widely used molluscicides is metaldehyde formulated as pelleted baits. These baits attract gastropods and upon ingestion are rapidly hydrolyzed to acetaldehyde, which causes the animal to produce excess mucus, dehydrate, and ultimately die (Triebskorn et al., 1998; Castle et al., 2017). However, these baits have variable efficacy due to a range of factors including weather conditions, different levels of attractiveness, and failure of a gastropod to consume enough bait (Crowell, 1967). Also, metaldehyde baits, along with most other molluscicides, are not targeted methods of pest control. Metaldehyde baits can harm a variety of different organisms including dogs, humans, and other organisms upon consumption (Castle et al., 2017). For example, ranking below chocolate ingestion, metaldehyde poisoning is the second most common cause of poisoning in canines (Cope et al., 2006). In mammals, metaldehyde is an irritant to the skin, eyes, mucous membranes, throat, and respiratory tract (Castle et al., 2017). The active ingredient may be leached at points of application and found in downstream river catchments at a level that can cause harm to non-target populations away from the application site (Gillman et al., 2012).

Parasitic nematodes within the genus *Phasmarhabditis* can be effective biological control agents against pestiferous gastropods with more targeted results compared to molluscicides (Rae et al., 2007). There are currently 16 nominal species of *Phasmarhabditis* worldwide (Wilson et al., 1993; Azzam, 2003; Huang et al., 2015; Nermut et al., 2016a,b; Tandingan De Ley et al., 2016; Ivanova and Spiridonov, 2017; Nermut et al., 2017; Pieterse et al., 2017; Ross et al., 2018; Pieterse, 2020; Zhang and Liu, 2020; Ivanova and Spiridonov, 2021). All species tested for their biological control potential have been shown to specifically target and kill gastropods, providing protection to a variety of crops (Wilson et al., 1993; Rae et al., 2007; Mc Donnell et al., 2018b,Mc Donnell et al., 2020; Nermut et al., 2020; Tandingan De Ley et al., 2020). *Phasmarhabditis hermaphrodita* is the most well-known and well-studied member of the genus. It is a facultative parasite that feeds on bacteria and can live saprobically or necromenically on gastropods or their feces (Tan and Grewal, 2001). *P. hermaphrodita* has seen success as a biological control agent across Europe as the commercially available product Nemaslug^®^. The species has undergone non-target testing with various species of earthworms, as well as native, non-pest European slugs and snails (Wilson et al., 2000; Grewal et al., 2003; Rae et al., 2005; Nardo et al., 2010). It did not cause mortality in any of the non-target species tested, suggesting that it is a safer alternative in Europe to traditional molluscicides, which are lethal to many organisms other than gastropods.

Until recently *Phasmarhabditis* had not been isolated within the United States. Therefore, due to agricultural policies, such as the National Environmental Policy Act (Montgomery, 2011), the commercialized Eurasian strain was not approved for use within the United States and as of 2021, it is still not commercially available in the United States. To find a viable biological control agent for gastropods in the United States, gastropod-nematode surveys within the country were performed to find a local species of nematode capable of causing mortality in slugs and snails. Three different surveys from 2000 to 2010 were performed to search for a gastropod biological control agent in the United States (Grewal et al., 2000; Kaya and Mitani, 2000; Ross et al., 2010). Most of the surveys looked for the presence of *Phasmarhabditis*, but they also searched for a variety of other nematode species found within gastropods and assessed their virulence. None of the surveys recovered any *Phasmarhabditis* species or other candidates for a gastropod biological control agent. However, over the past 8 years three species of *Phasmarhabditis* have been confirmed in California and one has been found in Oregon (Tandingan De Ley et al., 2014, 2016; Mc Donnell et al., 2018a). Thus, these local populations of *Phasmarhabditis* species should be the focus of future gastropod biological control research in the United States.

The first series of surveys which lead to the discovery of three *Phasmarhabditis* species in the United States in 2014 were conducted from 2012 to 2017 in California nurseries and garden centers. They were performed to search for potential biocontrol agents of invasive snails or slugs and to determine the distribution of parasitic nematodes including *Phasmarhabditis*. The species identified were *P. hermaphrodita*, *P. papillosa*, and a newly described species *P. californica* (Tandingan De Ley et al., 2014, 2016). As the next step, we evaluated the potential use of the local strains as biological control agents against invasive pestiferous gastropods in California (Tandingan De Ley et al., 2020).

Additional surveys were performed in 2018–2021 to determine the presence and distribution of gastropods and their associated *Phasmarhabditis* species, and to determine if the genus is widely established throughout the state. This series of extensive gastropod-nematode surveys is the first in the state of California. Such surveys have the potential to identify previously unknown nematode-gastropod relationships or identify new species of nematodes with biocontrol potential. These types of discoveries have been seen in other surveys performed across the globe (Ross et al., 2012; Tandingan De Ley et al., 2014; Mc Donnell et al., 2018a; Brophy et al., 2020). In this survey, we aimed to determine the presence and distribution of *Phasmarhabditis* nematodes and the diversity of gastropods in nurseries and garden centers throughout California.

## Materials and Methods

### Collection and Maintenance of Gastropods

We conducted gastropod surveys in 75 nurseries and garden centers during fall and winter months between 2012 and 2021 throughout California, covering at least 2 nurseries in each of the 28 counties surveyed. For ease of reference, the state was divided into three geographical areas: Northern California, Central California, and Southern California (Table 1 and Supplementary Figure 1). During the course of these surveys, 6,590 gastropod specimens were collected and brought back to the Insectary and Quarantine Facility and Departments of Entomology and Nematology at UC Riverside under CDFA Permits 2942 (2012–2018) and 3449 (2018–2022).

**TABLE 1:** Shows the California counties which were surveyed for gastropods and *Phasmarhabditis.*

2012–2017	2018–2021
Alameda	Butte
Fresno	Fresno
Humboldt	Humboldt
Kings	Kern
Madera	Los Angeles
Merced	Monterey
Monterey	Orange
Orange	Riverside
Plumas	San Bernardino
Riverside	San Diego
San Bernardino	San Luis Obispo
San Diego	Santa Barbara
San Luis Obispo	Santa Clara
San Mateo	Shasta
Santa Barbara	Sonoma
Santa Clara	Tehama
Santa Cruz	Tulare
Siskiyou	Ventura
Sonoma	
Stanislaus	
Tulare	
Ventura	
Yolo	

Gastropods were collected from nurseries and garden centers for a total of 1 person-hour per visit. For example, if 2 people were sampling, each person’s collection time would be 30 min. The gastropods were removed and collected from underneath potted plants, foliage, or plant trays on the ground using clean metal spatulas, and then immediately stored in plastic containers lined with moistened paper towels and covered with punctured lids (to maintain aeration). These containers were placed inside a cooler and at the end of each sampling day, the collected gastropods were sorted into 540 ml deli containers lined with a moistened paper towel and contained organic carrot pieces for food. The gastropods were sorted phenotypically by species, and the deli containers were labeled accordingly, and kept in coolers. Gastropods from different nurseries were kept in separate deli containers. The deli containers were cleaned every other day and were provided with a new moist paper towel and fresh organic carrot pieces. After each survey trip was completed, the gastropods were examined again to ensure they were identified correctly. In order to accurately identify gastropods, we used the methods described in Mc Donnell et al. (2009). We also had years of experience identifying California gastropods based off of the guide and received verification of our identifications by collaborating with gastropod expert Rory McDonnell. Once the gastropods were sorted correctly in the lab, relevant information was recorded and summarized, tracking the dates of collection, as well as the life history of the gastropods (e.g., when they were killed, viewed for infection, or whether they were infected). The gastropods were kept in the lab at room temperature with continued fresh changes of paper towel and organic carrot discs every other day. Each gastropod that died was given an accession number and immediately transferred to plated 1.1% plain agar (1 L: 10 g agar, 900 ml H_2_O) in order to obtain nematodes in seed culture, as described in Tandingan De Ley et al. (2014). To encourage better growth of nematodes, we modified the method and used nematode growth medium [NGM; 1 L: 3 g NaCl, 20 g Agar, 2.5 g Peptone, 975 ml deionized H_2_O, 10 ml Uracil (2 g/L) were added to a liter of deionized water, autoclaved, and let cool, to which were added 25 ml filtered KPO_4_, 1 ml filtered MgSO_4_, 1 ml CaCl_2_, and 1 ml Cholesterol 5 mg/ml)]. As surveys progressed in 2018, and in the interest of time, speed, and laboratory space, we modified our nematode recovery method, following the protocol of Wilson et al. (2016), i.e., decapitating slugs in batches and immediately placing them on NGM. This shortened our gastropod maintenance period, likely with the same outcome because gastropods infected with *Phasmarhabditis* were assumed to have harbored the nematode at the collection site. However, if *Phasmarhabditis* was transmitted within the laboratory, it is likely that the transmission only occurred across conspecifics collected at the same collection site since these gastropods were kept in the same container.

The gastropod-nematode surveys conducted between 2012–2017 and 2018–2021 were analyzed separately due to differences of collection time and survey methods. During the 2018–2021 survey, non-*Phasmarhabditis* nematodes were identified from host gastropods whereas this was not done in the 2012–2017 survey as a search for biocontrol candidates was targeted at finding *Phasmarhabditis* spp. and determining their distribution in California nurseries and garden centers. Each of the surveys also covered different counties throughout California, where the 2012–2017 survey often covered more nurseries and garden centers within each county, sometimes surveying the same nurseries multiple times. The 2018–2021 survey only surveyed each nursery once, and mostly covered two nurseries or garden centers per county (Table 1 and Supplementary Figure 1). While the methodology of collecting gastropods remained the same throughout each of the surveys, the separate analyses of the two allows for the assessment of gastropod diversity and abundance across time and allows for results to be interpreted upon each method.

### Nematode Recovery and Molecular Analyses

At least 5 individual nematodes that emerged from slug cadavers were picked from seed culture plates and grown on individual NGM plates, kept at 17°C. These plates of uniparental strains were labeled as single nematode isolations and were designated a unique accession number. Preliminary examination was done through a stereomicroscope, using morphological traits e.g., the presence of large phasmids and vulval body position, to identify suspected *Phasmarhabditis*. After suspects were identified, at least 2 individual nematodes from each single nematode isolation were prepared for PCR and DNA sequencing of the ribosomal RNA (D2-D3 domains of the large subunit or LSU), as described in Tandingan De Ley et al. (2014). When necessary, the small subunit (SSU) was also sequenced following the same protocols. Contigs were assembled and compared by BLAST with published sequences in GenBank using CodonCode Aligner (CodonCode Corp., 58 Beech Street, Dedham, MA, United States) to verify their identity or determine if sequences were unique.

## Results

### Gastropod Survey

**FIGURE 1:**
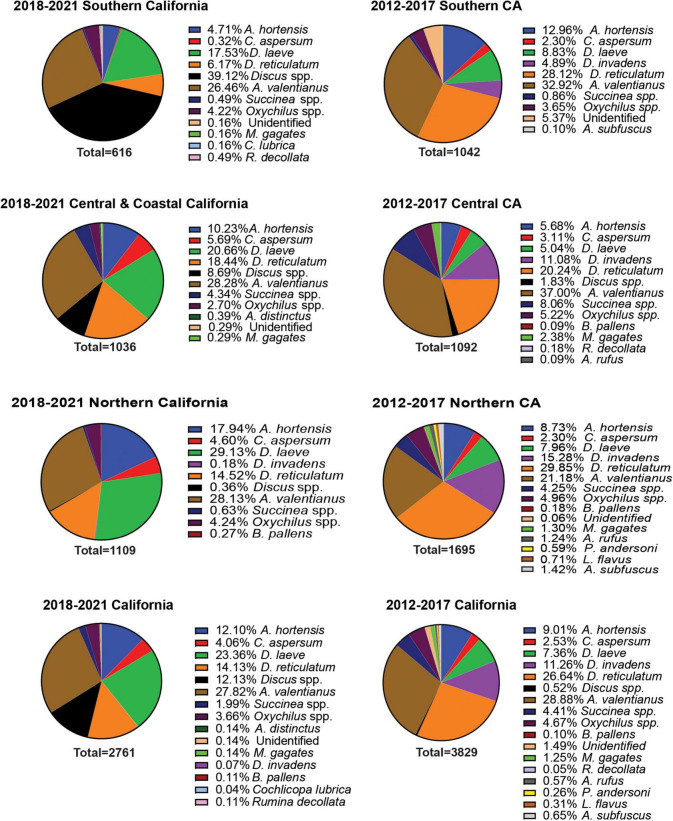
Percent recovery of terrestrial gastropods from different geographical regions of California during the 2012–2017 and 2018–2021 surveys. Surveys were performed during late fall or winter. Survey methods included 1 human hour searching for gastropods throughout each nursery. Collected gastropods were sorted by species and were taken back to the laboratory for later verification of species identity.

**FIGURE 2:**
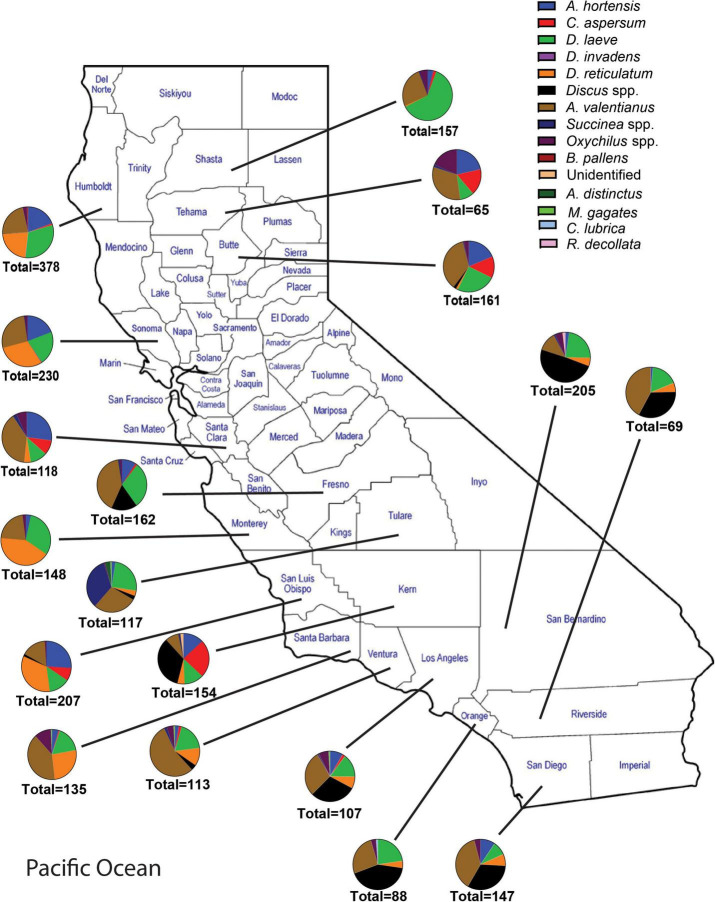
Abundance and species richness of terrestrial gastropods collected in each California county surveyed between 2018 and 2021. Surveys were performed during late fall or winter. Survey methods included 1 human hour searching for gastropods throughout each nursery. Collected gastropods were sorted by species and were taken back to the laboratory for later verification of species identity.

**FIGURE 3:**
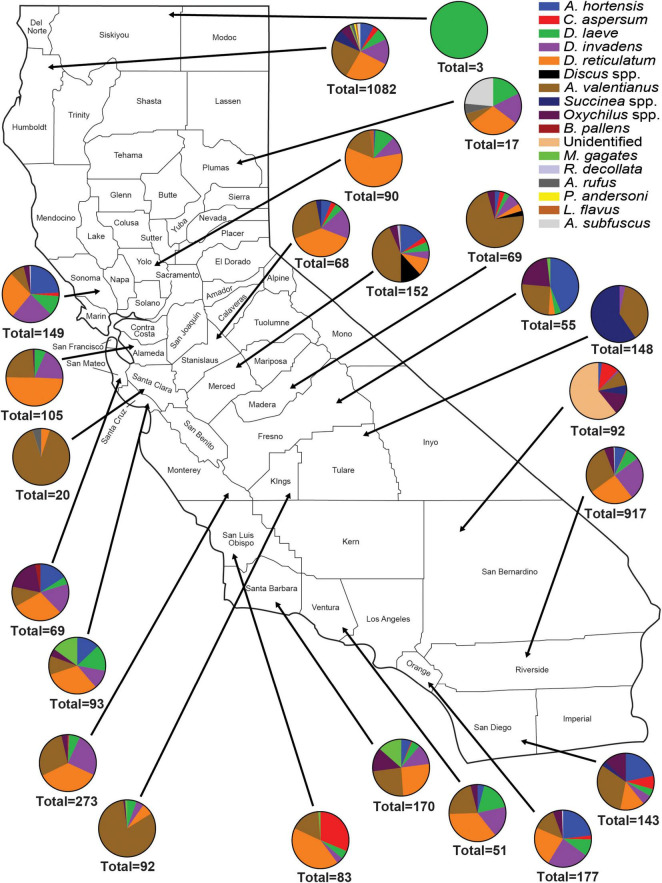
Abundance and species richness of terrestrial gastropods collected in nurseries in each California county surveyed between 2012 and 2017. Surveys were performed during late fall or winter. Survey methods included 1 human hour searching for gastropods throughout each nursery. Collected gastropods were organized by species and were taken back to the laboratory for later verification of species identity.

A total of 18 different gastropod species were recovered from all surveys. Sixteen of the 18 species recovered were invasive species, representing 99.8% of the total individuals collected (Figure 1). These include: *Arion hortensis* (Ferrusac 1819), *Arion distinctus* (Mabille 1869), *Arion rufus* (Linnaeus 1758), *Arion subfuscus* (Draparnaud 1805), *Cornu aspersum* (Müller 1774), *Deroceras laeve* (Müller 1774), *Deroceras reticulatum* (Müller 1774), *Deroceras invadens* (Reise et al., 2011), *Discus* spp., *Ambigolimax valentianus* (Ferussac 1821), *Sucinnea* spp., *Oxychilus* spp., *Milax gagates* (Lessona and Pollonera 1882), *Boettgerilla pallens* (Simroth 1912), *Cochlicopa lubrica* (Müller 1774), *Rumina decollata* (Linnaeus 1758), *Prophysaon andersoni* (Cockerell 1890), and *Limacus flavus* (Linnaeus 1758) (Figures 1–3). Both surveys from 2012 to 2017 and 2018 to 2021 recovered far more slug species (12) than snail species (6) (Figure 1). The two surveys, although completed over different years and with some differences between the counties visited and the nematodes which were chosen to be identified, were approximately congruent with a few notable disparities. The earlier survey obtained a greater number of *D. reticulatum* specimens in Southern California nurseries compared to the later survey (28.12% vs. 6.17%). *Discus* spp. were recorded during the later survey but were not collected during 2012–2017 (Figure 1). Also, the earlier gastropod surveys yielded a larger abundance of *D. invadens* across all areas of California. Each of the surveys also demonstrated that *A. valentianus* was the predominant gastropod species in nurseries. However, the second most common species collected during the 2012–2017 survey was *D. reticulatum*, while *D. laeve* was the second most common species during the 2018–2021 campaign. In general, more gastropod individuals were found at nurseries in Northern California than in other areas of California and fewer gastropod species were recovered in Southern California, indicating a possible decrease in gastropod abundance in a southward direction throughout the state (Figures 1–3).

### *Phasmarhabditis* Survey

A total of 69 *Phasmarhabditis* isolates were collected from all surveys. *Phasmarhabditis californica* was the most widespread species (37 isolates, 53.6% of all *Phasmarhabditis* recovered); followed by *P. hermaphrodita* (26 isolates; 37.7% recovery); *P. papillosa* and a *P. papillosa* closely related isolate (6 isolates; 8.7% recovery) (Table 2). The sequence of the D2-D3 expansion segment of 28S rDNA of this isolate was uploaded to Genbank (accession ID OL455007). Isolates were recovered from 5 invasive slug species: *D. reticulatum* (54%), *D. laeve* (25%), *A. hortensis* agg (5.7%), *A. valentianus* (8.7%) and two snails, *Oxychilus* spp. (5.8%) and *Discus* spp. (1.4%) (Table 2). Interestingly, isolates of *Phasmarhabditis* were mostly collected from *D. reticulatum* (53.6%), which was not the most abundant gastropod species found throughout the state. Only 8.7% of the isolates were collected from the most common gastropod, *A. valentianus* (Figures 1–3). However, about 78% of all isolates were collected from gastropod species within the genus *Deroceras* (Table 2).

**TABLE 2:** *Phasmarhabditis* species including hosts, sampling locations and morphological/genetic characterization from surveys performed between 2012 and 2021.

Nematode species	County	Host	Number of gastropods found with *Phasmarhabditis*
*P. hermaphrodita*			
	Alameda	*Deroceras reticulatum*	1
	Humboldt	*Ambigolimax valentianus*	1
	Humboldt	*Deroceras reticulatum*	2
	Monterey	*Deroceras laeve*	1
	Monterey	*Deroceras reticulatum*	6
	San Luis Obispo	*Deroceras laeve*	1
	San Luis Obispo	*Deroceras reticulatum*	10
	Santa Barbara	*Oxychilus* sp.	1
	Sonoma	*Deroceras laeve*	1
	Tehama	*Arion hortensis*	1
	Tulare	*Deroceras laeve*	1
*P. californica*			
	Alameda	*Deroceras reticulatum*	1
	Humboldt	*Arion hortensis*	1
	Humboldt	*Ambigolimax valentianus*	1
	Humboldt	*Deroceras laeve*	5
	Humboldt	*Deroceras reticulatum*	3
	Humboldt	*Oxychilus draparnaudi*	1
	Kern	*Discus* sp.	1
	Monterey	*Ambigolimax valentianus*	1
	Monterey	*Deroceras laeve*	1
	Monterey	*Deroceras reticulatum*	2
	Santa Clara	*Ambigolimax valentianus*	1
	Santa Clara	*Arion hortensis*	1
	San Luis Obispo	*Arion hortensis*	1
	San Luis Obispo	*Deroceras reticulatum*	2
	Santa Barbara	*Deroceras laeve*	5
	Santa Barbara	*Oxychilus draparnaudi*	2
	Sonoma	*Deroceras reticulatum*	3
	Tehama	*Deroceras reticulatum*	4
	Ventura	*Deroceras laeve*	1
*P. papillosa*			
	Los Angeles	*Ambigolimax valentianus*	2
	Los Angeles	*Deroceras laeve*	1
	Los Angeles	*Deroceras reticulatum*	1
	San Diego	*Deroceras reticulatum*	1
*Phasmarhabditis* sp.*			
	Monterey	*Deroceras reticulatum*	1

*Isolate closely related to Phasmarhabditis papillosa.

**FIGURE 4:**
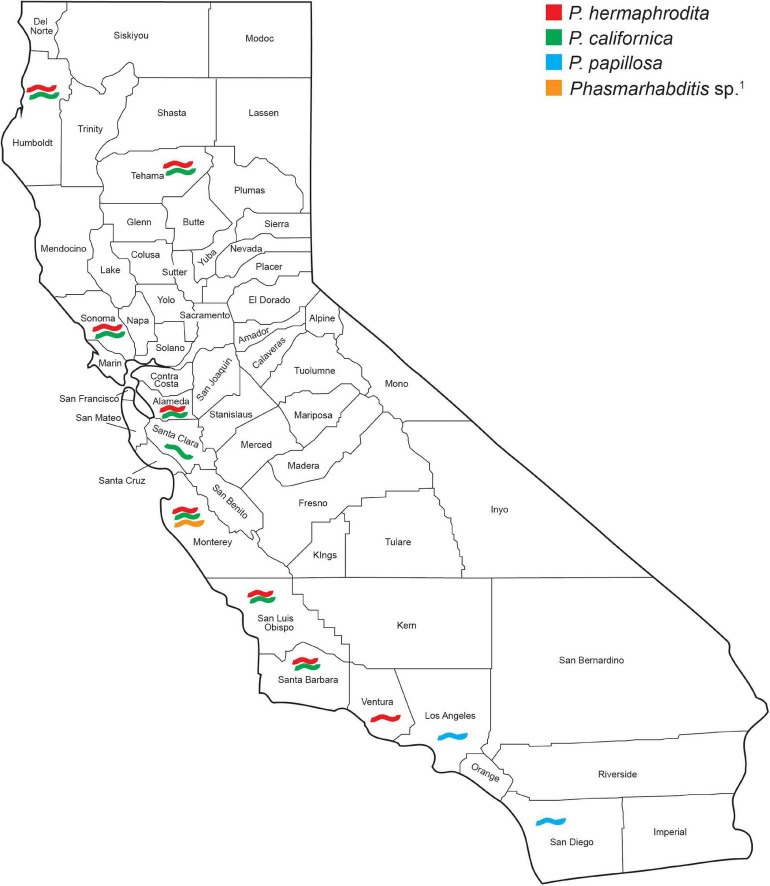
*Phasmarhabditis* species recovery and distribution among 28 California counties surveyed between 2012 and 2021. Species were identified by sequencing the D2–D3 expansion segments of the large subunit (LSU or 28S) ribosomal RNA and contigs compared by BLAST with published sequences in GenBank. ^1^Isolate closely related to *Phasmarhabditis papillosa*.

*Phasmarhabditis* isolates were collected and identified from Northern, Central, and Southern California. They were found in about 46% of all California counties surveyed. Results suggest that *P. californica* and *P. hermaphrodita* share an ecological niche throughout Northern CA and Central CA, whereas *P. papillosa* is mostly present by itself in Southern California. However, an unidentified close relative of *P. papillosa* was found in Monterey County (Central California) (Figure 4). Other nematode species were also recovered and identified from the surveys performed between 2018 and 2021. However, the non-*Phasmarhabditis* isolates are not representative of nematode diversity throughout California nurseries. This is because *Phasmarhabditis* was targeted, and only a select few nematodes from gastropod cadavers or seed cultures which did not morphologically resemble *Phasmarhabditis* were identified using 28S D2-D3 rDNA sequencing. Criteria for nematodes to be selected when they did not resemble *Phasmarhabditis* were not completely randomized. Nematodes which were not commonly observed (i.e. not a species of *Caenorhabditis*) were always selected for identification. Across locations, the most abundant non-*Phasmarhabditis* species identified was *Caenorhabditis elegans*, followed by *C. remanei* and *Rhabditophanes* spp. (Table 3). Other nematode species which are not typically considered to be associated with gastropods were also discovered. For example, *Cruzia americana*, a known opossum parasite, was discovered in a collected gastropod host (Li, 2019; Table 3). Some gastropod species that did not yield any associated *Phasmarhabditis* were found to have a variety of other associated nematode species (Table 4). *A. valentianus* had the most diverse nematode associations that included *A. dentiferum, Bursilla* spp., *C. elegans, C. remanei, C. tonkinensis*, and *Rhabditophanes* spp. (Table 4). However, this may well be the result of the larger sample size we obtained of *A. valentianus* compared to the other gastropod species. All nematode species identified can be found in Supplementary Table 1, as well as the host species they were discovered in. The locations in which all non-*Phasmarhabditis* nematodes were identified can be found in Supplementary Figure 2.

**TABLE 3:** Shows all recovered nematodes other than *Phasmarhabditis* during the California gastropod survey between 2018 and 2021.

Southern California		Central California		Northern California		California	
			
Nematode species	Number found	Nematode species	Number found	Nematode species	Number found	Nematode species	Number found
*Angiostoma dentiferum*	0	*Angiostoma dentiferum*	3	*Angiostoma dentiferum*	0	*Angiostoma dentiferum*	3
*Bursilla* spp.	1	*Bursilla* spp.	0	*Bursilla* spp.	0	*Bursilla* spp.	1
*Caenorhabditis elegans*	27	*Caenorhabditis elegans*	90	*Caenorhabditis elegans*	97	*Caenorhabditis elegans*	214
*Caenorhabditis remanei*	0	*Caenorhabditis remanei*	20	*Caenorhabditis remanei*	26	*Caenorhabditis remanei*	46
*Choriorhabditis cristata*	0	*Choriorhabditis cristata*	0	*Choriorhabditis cristata*	2	*Choriorhabditis cristata*	2
*Cosmocercoides pulcher*	0	*Cosmocercoides pulcher*	1	*Cosmocercoides pulcher*	0	*Cosmocercoides pulcher*	1
*Cosmocercoides tonkinensis*	11	*Cosmocercoides tonkinensis*	5	*Cosmocercoides tonkinensis*	0	*Cosmocercoides tonkinensis*	16
*Cuzia americana*	1	*Cuzia americana*	0	*Cuzia americana*	0	*Cuzia americana*	1
*Oscheius tipulae*	2	*Oscheius tipulae*	0	*Oscheius tipulae*	4	*Oscheius tipulae*	6
*Rhabditophanes* spp.	3	*Rhabditophanes* spp.	16	*Rhabditophanes* spp.	10	*Rhabditophanes* spp.	29

**TABLE 4:** Shows the species of nematodes (other than *Phasmarhabditis*) present in the cadavers of host gastropods found throughout gastropod surveys performed between 2018 and 2021.

*Arion hortensis*	*Ambigolimax valentianus*	*Cornu aspersum*	*Deroceras laeve*	*Deroceras reticulatum*
*Caenorhabditis elegans*	*Angiostoma dentiferum*	*Caenorhabditis elegans*	*Caenorhabditis elegans*	*Caenorhabditis elegans*
*Caenorhabditis remanei*	*Bursilla* spp.	*Caenorhabditis remanei*	*Caenorhabditis remanei*	*Caenorhabditis remanei*
*Oscheius tipulae*	*Caenorhabditis elegans*	*Rhabditophanes* spp.	*Cosmocercoides pulcher*	*Choriorhabditis cristata*
*Rhabditophanes* spp.	*Caenorhabditis remanei*		*Cosmocercoides tonkinensis*	*Cosmocercoides tonkinensis*
	*Cosmocercoides tonkinensis*		*Cruzia americana*	*Rhabditophanes* spp.
	*Oscheius tipulae*		*Rhabditophanes* spp.	
	*Rhabditophanes* spp.			

***Discus* spp.**	***Milax gagates***	***Oxychilus* spp.**	***Succinea* spp.**	

*Caenorhabditis elegans*	*Caenorhabditis elegans*	*Caenorhabditis elegans*	*Caenorhabditis elegans*	
*Caenorhabditis remanei*		*Caenorhabditis remanei*	*Choriorhabditis cristata*	
*Oscheius tipulae*		*Rhabditophanes* spp.		
*Rhabditophanes* spp.				

## Discussion

This was the first extensive gastropod and nematode survey performed throughout California. The surveys from 2012 to 2017 and 2018 to 2020 combined covered a total of 28 counties and resulted in the collection of 18 different gastropod species from 6,590 specimens. A total of 69 *Phasmarhabditis* isolates were collected. The most common gastropod species recovered was *A. valentianus*. According to this survey, invasive slug and snail species are more common than native gastropod species in California nurseries. Gastropod abundance decreased as we moved southward through California (Figures 1–3). This could be due to the desert and chapparal climates which occur in most of the southern sections of California, while Northern California climates include more precipitation. Slugs are prone to desiccation; therefore, lower survival rates in these climate conditions are plausible (Sternberg, 2000).

*Phasmarhabditis* species were found throughout all three geographic areas of California (Northern, Central, and Southern). Three species were identified throughout the state, *P. hermaphrodita*, *P. californica*, and *P. papillosa*. Also, one isolate was recovered in Monterey County which seems to be a close relative to or a variant of *P. papillosa* (Figure 4); with morphological characteristics diagnostic to the species. However, based on genetic analyses of the D2-D3 expansion segments of rRNA, it varies by 2 transitions, 1 ambiguity, 1 transversion, and 2 insertions/deletions (Thymine instead of Cytosine in nucleotide positions 46 and 67; C/T instead of Cytosine in position 140, Adenine instead of a Cytosine in position 261, and 2 indels on positions 346 and 347, respectively). Surveys in Oregon showed the same 3 *Phasmarhabditis* species, and interestingly, *P. hermaphrodita* was recovered from a slug in a *Brassica* field in Salem, OR, suggesting it may also be present in the wider agricultural environment (Howe et al., 2020). In California, our surveys focused solely on nurseries and garden centers because (1) horticulture is one of the most valuable agricultural industries in the state (2) slugs and snails are major pests in the industry and (3) transportation of plants to and from retail nurseries presumably causes gastropods to be moved around the state over great distances than would otherwise be possible. The nurseries themselves could therefore be focal locations for exposure of invasive slug and snail species to a greater diversity of gastropod associated nematodes than is likely to occur in production fields or greenhouses. Based on the Oregon finding, it is likely that these gastropod-infecting nematodes may have also found their way as hitchhikers into agricultural and horticultural fields and backyard gardens in California. However, that has yet to be determined as these production areas were not covered in our surveys.

Additionally, recent studies based on mitochondrial DNA COI gene phylogenies, showed that *Phasmarhabditis hermaphrodita* U.S. isolates and strains (including isolates from the CA 2012 to 2017 survey and OR surveys) had haplotypes that were nearly identical to *P. hermaphrodita* collected in the United Kingdom for commercialization of Nemaslug^®^. They were placed together in an intraspecific monophyletic clade with the Nemaslug^®^ strain (Howe et al., 2020). We can hypothesize that *P. hermaphrodita* found in the United States likely came from areas where Nemaslug^®^ was used in Europe. Invasion of California and Oregon probably came about from agricultural trade with interstate movement of infected soil and/or slugs/snails. The invasive slugs from Europe and some of the specimens found in this survey likely came with these same nematodes of near-identical haplotypes. It is equally likely that the nematodes came to California on slugs many years ago before the commercialization of Nemaslug^®^, and they have stayed in the region by infecting *Deroceras* slugs and other suitable host pest slugs which are now established in California. In the study by Howe et al. (2020), available *P. californica* strains at that time were also studied. As with *P. hermaphrodita*, all *P. californica* haplotypes (CA, United States; United Kingdom; and New Zealand) belonged to one single, strongly supported clade. Interestingly, *P. californica* shares the same geographical niche and host gastropod species as *P. hermaphrodita* from Northern to Southern California. However, *P. papillosa* seems to only inhabit areas of Southern California (Figure 4).

Some gastropod species were more commonly infected with *Phasmarhabditis* than others. The gastropod host *A. valentianus*, which was the most frequently found gastropod, only accounted for about 8.5% of the *Phasmarhabditis* isolates collected. *A. valentianus* may have a more developed immune response to parasitic nematodes compared to other slugs, however, this has yet to be determined. The majority of *Phasmarhabditis* nematodes collected from the survey were collected from *D. reticulatum*. The host *D. reticulatum* accounted for about 55% of all *Phasmarhabditis* nematodes collected. In total, the genus *Deroceras* accounted for about 74.1% of the identified *Phasmarhabditis* nematodes (Table 2). *D. reticulatum* is a common slug pest across Europe, especially in areas near Ireland and the United Kingdom where Nemaslug^®^ was originally discovered (Kerney, 1999). This serves as additional evidence that an infected invasive species of gastropod from Europe likely brought *Phasmarhabditis* to the United States where the relationship between the gastropod hosts remained.

Multiple gastropods collected throughout the survey were infected and/or associated with a diverse array of nematodes (other than *Phasmarhabditis*) (Supplementary Table 1 and Tables 3, 4). *C. elegans* and *C. remanei* were the most prominent nematodes found within gastropod hosts. These nematodes are not uncommon in gastropods, and though the interaction between *C. remanei* and gastropods has not been thoroughly explored, *C. elegans* is thought to have a phoretic association with gastropods (Caswell-Chen et al., 2005; Ross et al., 2012; Petersen et al., 2015; Rae, 2017; Sudhaus, 2018). *C. elegans* is also known to have phoretic associations with some species of earthworms and arthropods (Kiontke and Sudhause, 2006; Brophy et al., 2020). Other interesting nematode species were also discovered during the California surveys including *Cosmocercoides tonkinensis*, which is not commonly associated with gastropod hosts (Supplementary Table 1 and Tables 3, 4; Sudhaus, 2018). *C. tonkinensis* has only been described in reptiles (Tran et al., 2015). However, for another member of the genus, *Cosmocercoides dukae*, mollusks are a known host (Anderson, 1960). A survey done in 2014 which identified *P. hermaphrodita* in California also recovered species other than *Phasmarhabditis* within gastropod hosts. Some of these species included *Alloionema appendiculatum* (a common parasite of slugs), *C. elegans*, *C. briggsae*, a new species *A. similis*, and species of *Oscheius* (Tandingan De Ley et al., 2014; Holovachov et al., 2016). Our non-*Phasmarhabditis* results share in some of these genus recoveries, except for *Alloionema* spp. (Table 3). The absence of this genus throughout both surveys spanning from 2012 to 2021 is unexpected and intriguing since it was discovered in past surveys in similar locations (Laznik et al., 2010).

The occurrence of *P. hermaphrodita* and other species of the genus in North America has regulatory implications for potential biocontrol strategies against non-native slug and snail species that are pests of agriculture on this continent. Since the nematode occurs throughout the state, its use in a similar manner to Nemaslug^®^ may be a feasible option. Its use could potentially save the California specialty crop industry about 64 million dollars, and is therefore worth exploring as a biological control option (Supplementary Table 2). The recovery of *Phasmarhabditis* from local plant nurseries and garden centers throughout California was not entirely surprising as these are considered transport hubs for non-native gastropod species (Bergey et al., 2014). It is not known if *Phasmarhabditis* exists in the natural environment throughout California where horticultural practices do not take place. In order to better understand the presence of *Phasmarhabditis* in the state, further surveillance is required in horticultural and agricultural field production areas, as well as natural ecosystems. Also, additional non-target and target host experiments with *Phasmarhabditis* are required to have a deeper understanding of how these potential biological control agents will affect the local ecosystem where they would likely be introduced. Additionally, host experimentation should be performed in mesocosms or other field-like conditions to determine efficacy.

## Data Availability Statement

The datasets presented in this study can be found in online repositories. The names of the repository/repositories and accession number(s) can be found below: Mendeley Data, V2, doi: 10.17632/dwxh3bmmcb..

## Author Contributions

JS carried out nematode studies and identification, conceptualized, planned, and carried out the survey, identified gastropods, maintained gastropods and nematodes and created records for all specimens, performed nematode genetic identification work, and wrote and designed the manuscript. ITD conceptualized, planned, carried out, and supervised the nematode genetic studies and identification, secured fundings, performed the survey for gastropods and nematodes throughout California, and assisted in the writing and development of the manuscript. KA assisted in genetic identification and maintenance of gastropods and nematodes. TP provided guidance and secured funding throughout early stages of the project. RM performed survey for all gastropods throughout California between 2012 and 2017, assisted in gastropod identification during the surveys between 2018 and 2020, secured funding, and assisted in the development of the manuscript. AD secured funding, managed the project, and assisted in the development of the manuscript. All authors contributed to the article and approved the submitted version.

## Conflict of Interest

ITD, RM, and TP, declare that they are co-inventors on a patent entitled Mollusk-killing Biopesticide (WO2017059342A1). The remaining authors declare that the research was conducted in the absence of any commercial or financial relationships that could be construed as a potential conflict of interest.

## Publisher’s Note

All claims expressed in this article are solely those of the authors and do not necessarily represent those of their affiliated organizations, or those of the publisher, the editors and the reviewers. Any product that may be evaluated in this article, or claim that may be made by its manufacturer, is not guaranteed or endorsed by the publisher.

## Supplementary Material

Supplementary Figure 1 California counties (28) surveyed for terrestrial gastropods and gastropod-associated nematodes from 2012 to 2021.

Supplementary Figure 2 Locations which non-*Phasmarhabditis* were found throughout the gastropod surveys conducted between 2018 and 2021.
