# Supplementary material for: Distribution of Phasmarhabditis (Nematode: Rhabditidae) and Their Gastropod Hosts in California Plant Nurseries and Garden Centers
Source: Front Plant Sci. 2022 May 17;13:856863. doi: 10.3389/fpls.2022.856863 (PMC9152542; doi:10.3389/fpls.2022.856863)
Supplement: Supplementary file 1 [file Table_1.docx]

**Table S1.** Shows all nematode species identified and the gastropods they were discovered in during the 2018-2021 survey.

| **Nematode/Gastropod Species** | **Northern California** |  | **Nematode/Gastropod Species** | **Central California** |  | **Nematode/Gastropod Species** | **Southern California** |  | **Total** |  |
| --- | --- | --- | --- | --- | --- | --- | --- | --- | --- | --- |
| ***Angiostoma dentiferum*** | x |  | ***Angiostoma dentiferum*** | 3 |  | ***Angiostoma dentiferum*** | x |  | ***Angiostoma dentiferum*** | 3 |
| x | x |  | *Ambigolimax valentianus* | x |  | x | x |  | *Ambigolimax valentianus* |  |
| ***Bursilla sp.*** | x |  | ***Bursilla spp.*** | x |  | ***Bursilla spp.*** | 1 |  | ***Bursilla spp.*** | 1 |
| x | x |  | x | x |  | *Ambigolimax valentianus* |  |  | *Ambigolimax valentianus* |  |
| ***C. elegans*** | 97 |  | ***C. elegans*** | 90 |  | ***C. elegans*** | 27 |  | ***C. elegans*** | 214 |
| *Arion hortensis* |  |  | *Arion hortensis* |  |  | *Arion hortensis* |  |  | *Arion hortensis* |  |
| *Ambigolimax valentianus* |  |  | *Ambigolimax valentianus* |  |  | *Ambigolimax valentianus* |  |  | *Ambigolimax valentianus* |  |
| *Cornu aspersum* |  |  | *Cornu aspersum* |  |  | *Deroceras reticulatum* |  |  | *Cornu aspersum* |  |
| *Deroceras laeve* |  |  | *Deroceras laeve* |  |  | *Discus spp.* |  |  | *Deroceras laeve* |  |
| *Deroceras reticulatum* |  |  | *Deroceras reticulatum* |  |  | *Succinea spp.* |  |  | *Deroceras reticulatum* |  |
| *Milax Gagates* |  |  | *Discus spp.* |  |  | x |  |  | *Discus spp.* |  |
| *Oxychilus spp.* |  |  | *Succinea spp.* |  |  | x |  |  | *Milax gagates* |  |
| ***C. remanei*** | 26 |  | ***C. remanei*** | 20 |  | ***C. remanei*** | x |  | *Oxychilus spp.* |  |
| *Arion hortensis* |  |  | *Ambigolimax valentianus* |  |  | x |  |  | *Succinea spp.* |  |
| *Ambigolimax valentianus* |  |  | *Deroceras laeve* |  |  | x |  |  | ***C. remanei*** | 46 |
| *Cornu aspersum* |  |  | *Deroceras reticulatum* |  |  | x |  |  | *Arion hortensis* |  |
| *Deroceras laeve* |  |  | *Discus spp.* |  |  | x |  |  | *Ambigolimax valentianus* |  |
| *Deroceras reticulatum* |  |  | *Oxychilus spp.* |  |  | x |  |  | *Cornu aspersum* |  |
| *Oxychilus spp.* |  |  | x |  |  | x |  |  | *Deroceras laeve* |  |
| ***Choriorhabditis cristata*** | 2 |  | ***Choriorhabditis cristata*** | 5 |  | ***Choriorhabditis cristata*** | x |  | *Deroceras reticulatum* |  |
| *Deroceras reticulatum* |  |  | x |  |  | x |  |  | *Discus spp.* |  |
| *Succinea spp.* |  |  | x |  |  | x |  |  | *Oxychilus spp.* |  |
| ***Cosmocercoides pulcher*** | x |  | ***Cosmocercoides pulcher*** | x |  | ***Cosmocercoides pulcher*** | x |  | ***Choriorhabditis cristata*** | 2 |
| x |  |  | *Deroceras laeve* |  |  | x |  |  | *Deroceras reticulatum* |  |
| ***Cosmocercoides tonkinensis*** | x |  | ***Cosmocercoides tonkinensis*** | x |  | ***Cosmocercoides tonkinensis*** | 11 |  | *Succinea spp.* |  |
| x |  |  | *Deroceras laeve* |  |  | *Ambigolimax valentianus* |  |  | ***Cosmocercoides pulcher*** | 1 |
| x |  |  | x |  |  | *Deroceras laeve* |  |  | *Deroceras laeve* |  |
| x |  |  | x |  |  | *Deroceras reticulatum* |  |  | ***Cosmocercoides tonkinensis*** | 16 |
| ***Cruzia americana*** | x |  | ***Cruzia americana*** | x |  | ***Cruzia americana*** | 1 |  | *Ambigolimax valentianus* |  |
| x |  |  | x |  |  | *Deroceras laeve* |  |  | *Deroceras laeve* |  |
| ***Oscheius tipulae*** | 4 |  | ***Oscheius tipulae*** | x |  | ***Oscheius tipulae*** | 2 |  | *Deroceras reticulatum* |  |
| *Arion hortensis* |  |  | x |  |  | *Discus spp.* |  |  | ***Cruzia americana*** | 1 |
| *Ambigolimax valentianus* |  |  | x |  |  | x |  |  | *Deroceras laeve* |  |
| ***Rhabditophanes*** | 10 |  | ***Rhabditophanes*** | 16 |  | ***Rhabditophanes*** | 3 |  | ***Oscheius tipulae*** | 6 |
| *Ambigolimax valentianus* |  |  | *Arion hortensis* |  |  | *Ambigolimax valentianus* |  |  | *Arion hortensis* |  |
| *Cornu aspersum* |  |  | *Ambigolimax valentianus* |  |  | *Discus spp.* |  |  | *Ambigolimax valentianus* |  |
| *Deroceras laeve* |  |  | *Cornu aspersum* |  |  | *Oxychilus spp.* |  |  | *Discus spp.* |  |
| *Oxychilus spp.* |  |  | *Deroceras laeve* |  |  | x |  |  | ***Rhabditophanes*** | 29 |
| x |  |  | *Deroceras reticulatum* |  |  | x |  |  | *Arion hortensis* |  |
|  |  |  |  |  |  |  |  |  | *Ambigolimax valentianus* |  |
|  |  |  |  |  |  |  |  |  | *Cornu aspersum* |  |
|  |  |  |  |  |  |  |  |  | *Deroceras laeve* |  |
|  |  |  |  |  |  |  |  |  | *Deroceras reticulatum* |  |
|  |  |  |  |  |  |  |  |  | *Discus spp.* |  |
|  |  |  |  |  |  |  |  |  | *Oxychilus spp.* |  |
