# Supplementary material for: Distribution of Phasmarhabditis (Nematode: Rhabditidae) and Their Gastropod Hosts in California Plant Nurseries and Garden Centers
Source: Front Plant Sci. 2022 May 17;13:856863. doi: 10.3389/fpls.2022.856863 (PMC9152542; doi:10.3389/fpls.2022.856863)
Supplement: Supplementary file 2 [file Table_2.DOCX]

|  | **California Specialty Crops** | |
| --- | --- | --- |
| **Agriculture** | **Number of Farms** | **Sales** |
| Nurseries, Greenhouses, Floriculture, and Sod | 3,890,000 | $2,547,307,000 |
| Assumed 5% Loss to Gastropod Damage |  | $127,365,350 |
| Potential Gain from *Phasmarhabditis* Use |  | $64,166,663 |

**Table S2**. Shows the estimated return of investment (ROI) of using *Phasmarhabditis* on California specialty crops which are frequently affected by gastropod pests. These crops include those found in nurseries, greenhouses, floriculture, and sod industries. Data assumes a mean damage reduction of 50.38% based on Rae *et al*., 2007. ROI chart was made based off chart made and presented by Irma Tandingan De Ley, 2017 (unpublished). Sourced information comes from the 2012 Census of Agriculture for Specialty Crops vol. 2 part 8 (published February 2015): https://agcensus.usda.gov/Publications/2012/Online_Resources/Specialty_Crops/SCROPS.pdf
