# Supplementary figures and images for: Distribution of Phasmarhabditis (Nematode: Rhabditidae) and Their Gastropod Hosts in California Plant Nurseries and Garden Centers
Source: Front Plant Sci. 2022 May 17;13:856863. doi: 10.3389/fpls.2022.856863 (PMC9152542; doi:10.3389/fpls.2022.856863)

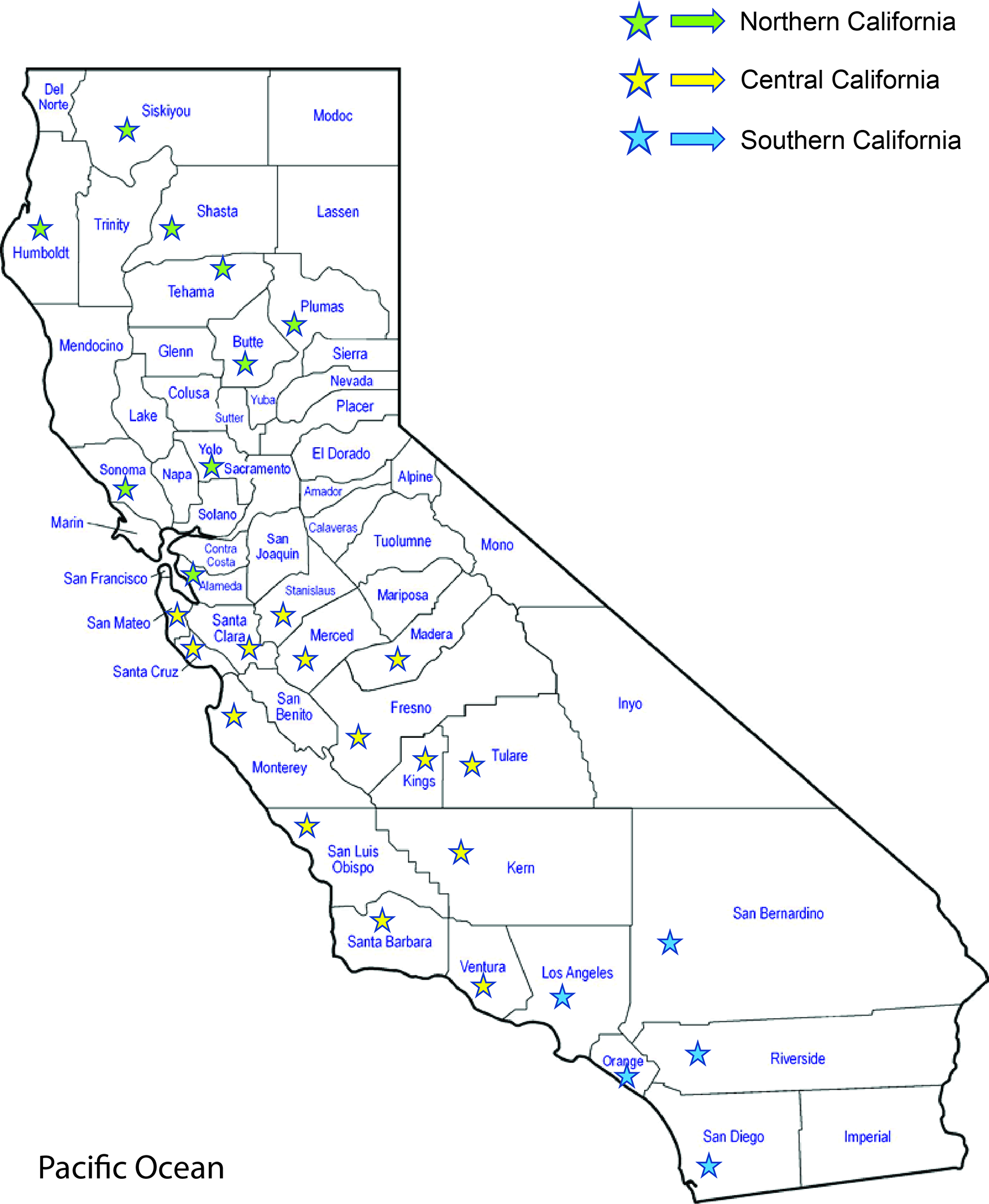

Supplement: Supplementary Figure 1 — California counties (28) surveyed for terrestrial gastropods and gastropod-associated nematodes from 2012 to 2021. [file Image_1.tif]

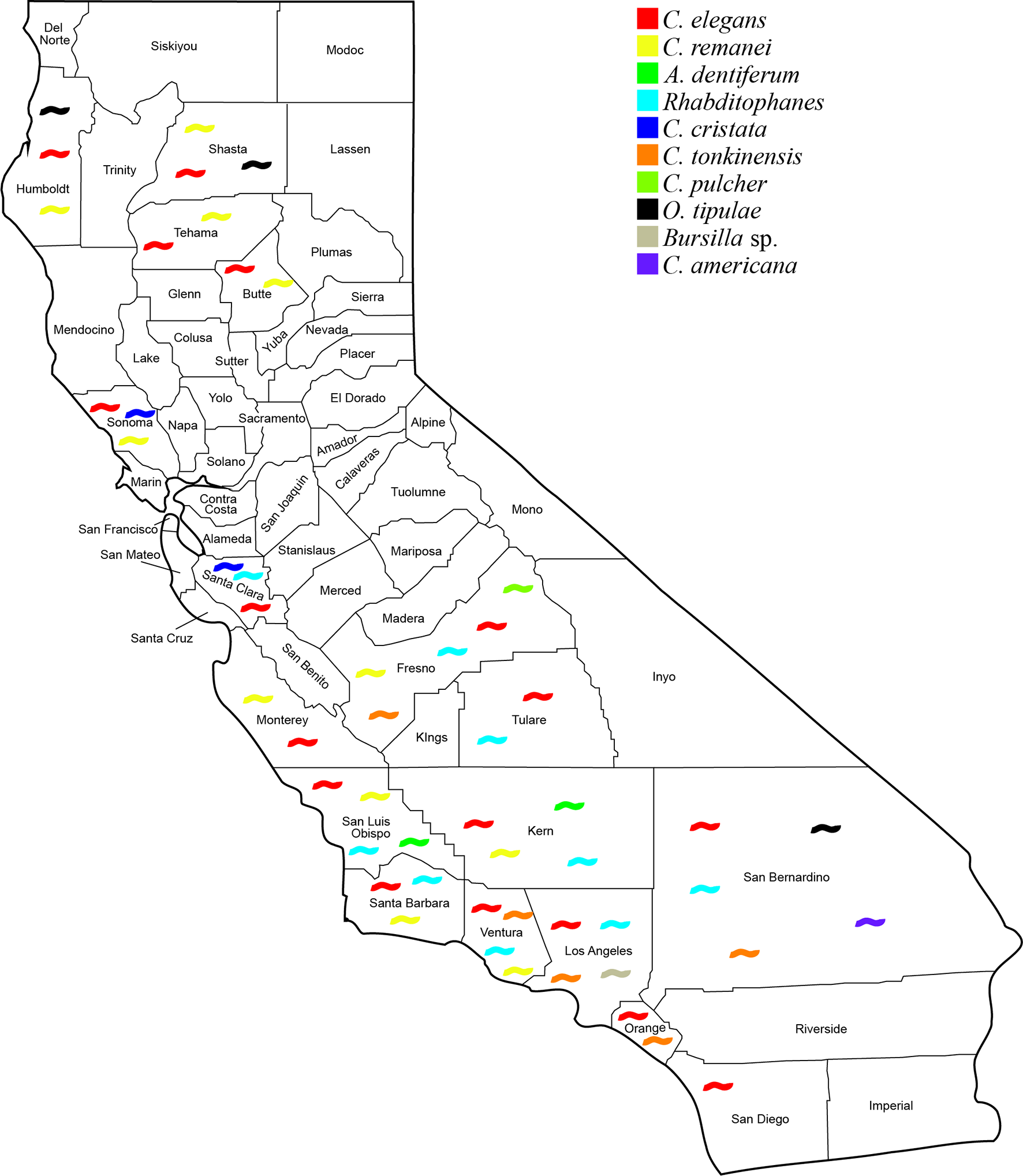

Supplement: Supplementary Figure 2 — Locations which non-Phasmarhabditis were found throughout the gastropod surveys conducted between 2018 and 2021. [file Image_2.TIF]
